# Adhesion of monocytes to type I collagen stimulates an APP-dependent proinflammatory signaling response and release of Aβ1-40

**DOI:** 10.1186/1742-2094-7-22

**Published:** 2010-03-19

**Authors:** Cindy M Sondag, Colin K Combs

**Affiliations:** 1 Department of Pharmacology, Physiology & Therapeutics, University of North Dakota School of Medicine and Health Sciences, 504 Hamline St., Room 118, Grand Forks, ND 58203, USA

## Abstract

**Background:**

Amyloid precursor protein (APP) is a ubiquitously expressed cell surface protein reported to be involved in mediating cell-cell or cell-matrix interactions. Prior work has demonstrated that APP co-localizes with β1 integrin in different cell types.

**Methods:**

In an effort to determine the function of APP on monocytic lineage cells, in particular, the human monocyte cell line, THP-1, was used to assess the role of APP during adhesion to the extracelluar matrix component type I collagen.

**Results:**

Pull-down assays demonstrated that THP-1 adhesion to collagen stimulated a tyrosine kinase-associated signaling response which included subsequent phosphorylation of p38 MAP kinase and increased association of APP with α2β1 integrin, specifically. In addition, cell adhesion was dependent upon APP expression since APP siRNA knockdown attenuated THP-1 adhesion to collagen compared to mock transfected controls. One consequence of the tyrosine kinase-dependent signaling response was increased secretion of interleukin-1β (IL-1β) and Aβ1-40 but not the Aβ1-42 fragment of APP. Increased secretion of IL-1β was dependent upon p38 MAP kinase activity while Aβ1-40 secretion required Src family kinase activity since the specific p38 inhibitor, SB202190, and the Src family kinase inhibitor, PP2, attenuated IL-1β and Aβ1-40 secretion, respectively.

**Conclusions:**

These data demonstrate that APP is involved in classic integrin-dependent tyrosine kinase-associated adhesion and activation of peripheral monocytic cells. Moreover, divergent APP-dependent signaling is required for increased secretion of both IL-1β and Aβ1-40 as a component of the adhesion-dependent change in phenotype. This suggests that APP may have a broad role in not only mediating cell-matrix adhesion but also in the function of peripheral immune cells.

## Background

The progressive accumulation of extracellular beta amyloid-containing plaques is a cardinal feature of Alzheimer's disease (AD) pathology. The beta amyloid (Aβ) peptide is generated through sequential cleavage of the amyloid precursor protein (APP) by β- and γ-secretase. Altered proteolysis of APP due to missense mutations in the APP gene results in increased production of Aβ and increased accumulation of plaques [1-3]. APP is a ubiquitously expressed integral membrane protein with a relatively undefined function separate from generating Aβ [4]. Several data support a role for APP in adhesion including evidence that APP co-localizes with β1 integrins in both neurons and monocytes as well as evidence that APP binds directly to type I collagen and other extracellular matrix (ECM) molecules [5-8].

Adhesion of monocytes to vascular endothelium is a highly regulated, necessary step in transendothelial migration that occurs constitutively but also during a variety of conditions including peripheral inflammation, infection, atherosclerosis, neurodegenerative disease, and injury [9-14]. Monocyte adhesion to the endothelium initiates changes in their gene expression important in differentiation and extravasation [12]. During diapedesis, monocytes also closely interact with extracellular matrix components, in part through integrin receptor interaction, which can further activate these cells towards macrophage differentiation [15-17]. Integrin-mediated activation of monocytes is characterized by a tyrosine-kinase dependent proinflammatory signaling response and release of multiple proinflammatory molecules [7,18-21]. Interestingly, *in vitro *exposure of endothelial cells to Aβ1-40 up-regulates endothelial adhesion molecule expression and monocytic adhesion to these cells, thus facilitating the transmigration process [22].

Plasma levels of Aβ have debatable implications with regard to increased risk for cerebral amyloid angiopathy [23]. Aβ1-40, rather than Aβ1-42, appears to be the dominant component in the vascular depositions [24,25]. While Aβ1-42 levels and deposition in the brain are correlated with the incidence of AD, it has been demonstrated that increased levels of plasma Aβ1-40 correlate with an increased incidence of dementia [26]. Platelets are a known source of peripheral Aβ production and recent data shows an increased β-secretase activity in platelets from patients with AD [27,28]. However, monocytes are another peripheral source of Aβ and interestingly it has been shown that adhesion stimulates an increased release of Aβ1-40 from these cells [29].

This study aims to further characterize the role of APP in adhesion-mediated activation of monocytes. In addition, we propose that β1 integrin ligation of monocytes by an endogenous ligand such as type I collagen is an important mechanism through which peripheral Aβ is released, and this may have implications in pathological accumulations of Aβ in the brain parenchyma as well as in the vasculature.

## Methods

### Materials

The 4G10 monoclonal anti-phosphotyrosine antibody was purchased from Upstate Biotechnology (Lake Placid, NY). Anti-β-Amyloid Precursor Protein polyclonal antibody was from Zymed Laboratories (San Francisco, CA). Anti-phospho-p38, anti-p38, anti-phospho-JNK, anti-JNK were from Cell Signaling (Beverly, MA). Anti-α2 integrin antibody, anti-α1 integrin antibody, anti-β1 integrin antibody, anti-phospho-ERK, anti-ERK2 antibody, and Protein A/G PLUS-Agarose beads were from Santa Cruz Biotechnology (Santa Cruz, CA). The 22C11 anti-APP monoclonal antibody, gamma-secretase inhibitor, and beta-secretase inhibitor were from Calbiochem (La Jolla, CA). Anti-mouse (goat), anti-rabbit (donkey), and anti-goat (donkey) horseradish peroxidase (HRP) conjugated secondary antibodies were purchased from Santa Cruz Biotechnology (Santa Cruz, CA). APP siRNA (siGene, SMARTpool Plus) was obtained from Dharmacon (Lafayette, CO). Type I collagen was derived from rat tail following a standard protocol. All other materials not specified were purchased from Sigma (St. Louis, MO).

### Tissue culture

THP-1 cells are a monocytic cell line derived from peripheral blood of a human with acute monocytic leukemia commercially available from the American Type Culture Collection (Manassas, VA). THP-1 cells were grown in RPMI-1640 (Gibco RBL, Rockville, MD) containing 10% heat inactivated fetal bovine serum (FBS) (U.S. Biotechnologies Inc., Parkerford, PA), 5 mM Hepes, and 1.5 μg/mL pen/strep/neomycin.

### Cell stimulation

THP-1 monocytes were removed from normal growth medium into serum-free RPMI and incubated at 37°C for fifteen minutes prior to stimulation. Tissue culture wells were coated with type I rat tail collagen and allowed to dry in a laminar flow hood. THP-1 cells were added to either low-adhesion tissue culture plastic alone or to wells coated with type I collagen at a density of 2 × 10^6 ^cells/mL. Cells were stimulated for either 30 min, 24 hours, or 48 hours. In inhibition studies, cells were incubated at 37°C for 30 minutes with inhibitors before stimulation.

### Western blotting

Ice cold RIPA buffer (20 mM Tris pH 7.4, 150 mM NaCl, 1 mM Na_3_VO_4_, 10 mM NaF, 1 mM EDTA, 1 mM EGTA, 0.2 mM PMSF, 1% Triton, 0.1% SDS, 0.5% deoxycholate) was used to lyse cells. Cell lysates were sonicated and centrifuged (14,000 RPM, 4°C, 10 min) to remove insoluble material. Protein concentrations were quantitated by the method of Bradford [30]. Proteins were resolved by 7% or 10% SDS-PAGE and transferred to PVDF membranes for Western blotting. Western blots were blocked in TBS-T (10 mM Tris, pH 7.4, 100 mM NaCl, and 0.1% Tween 20) containing 3% BSA for 15 minutes and then incubated overnight at 4°C in primary antibodies. Blots were washed three times in TBS-T, followed by incubation for one hour with HRP conjugated secondary antibodies in TBS-T containing 5% nonfat dried milk. The blots were washed three times in TBS-T followed by detection with enhanced chemiluminescence (Pierce, Rockford, IL). In some instances, blots were stripped in 0.2 N NaOH, 5 minutes, 25°C.

### Immunoprecipitation

For co-immunoprecipitation, cells were lysed in ice cold Triton lysis buffer (20 mM Tris pH 7.4, 150 mM NaCl, 1 mM Na_3_VO_4_, 10 mM NaF, 1 mM EDTA, 1 mM EGTA, 0.2 mM PMSF, 1% Triton). Lysates were vortexed then incubated on ice for 15 minutes followed by pulse sonication. Cells were centrifuged 10 minutes at 4°C to remove insoluble material. Primary antibody (1 μg/mg protein) was added and incubated 2 hours at 4°C. Protein A/G beads (35 μl) were added and incubated 2 hours at 4°C. Beads were washed three times with lysis buffer and immunoprecipitates were resolved and Western blotted as described above.

### Kinase and secretase Inhibition

4-amino-5-(4-chlorophenyl)-7-(*t*-butyl)pyrazolo [3,4-*d*]pyrimidine (PP2) was purchased from Alexis Biochemicals (San Diego, CA). PP2 is a small-molecule inhibitor specific for Src-family kinases believed to be a mixed competitive inhibitor vis-à-vis the substrate [31]. The cell permeable pyridinyl imidazole compound, SB202190, inhibits p38 kinase activity through competition with ATP binding (Calbiochem) [32]. The gamma-secretase inhibitor, Z-Leu-Leu-Nle-CHO, is a cell permeable synthetic tripeptide aldehyde inhibitor of gamma secretase activity (Calbiochem) [33]. The beta secretase inhibitor, Z-VLL-CHO; N-Benzyloxycarbonyl-val-leu-leucinal is a cell permeable, reversible inhibitor (Calbiochem) [34].

### Adhesion assay

THP-1 cells were loaded with calcein-AM (Molecular Probes, Eugene, OR) by incubating at 1 × 10^6 ^cells/mL in serum-free RPMI 1640 medium containing 5 μg/mL calcein-AM for 30 min at 37°C. Cells were washed three times with medium and resuspended at a plating density of 1 × 10^6 ^cells/mL. 50 μl aliquots were added to a 96-well plate coated with collagen and incubated at 37°C and 5% CO_2 _for 45 min. Total cell number per well was then assessed by reading the plates at an excitation wavelength of 485 nm and an emission wavelength of 538 nm prior to washing the wells. Plates were then washed three times with PBS and fluorescence of adhered cells was determined at an excitation wavelength of 485 nm and an emission wavelength of 538 nm. The fluorescence value of adhered cells was then normalized according to the original cell number value.

### Enzyme linked-immuno-sorbent assay (ELISA)

Media was collected from THP-1 cells following 24 hour or 48 hour stimulation. Media was centrifuged for clarification (14,000 × g, 2 min, 25°C) prior to analysis. Levels of human interleukin-1β, Aβ1-42, and Aβ1-40 in the media were determined using commercially available ELISA kits (Immuno-Biological Laboratories Inc. Minneapolis, MN), according to the manufacturer's protocol.

### APP siRNA transfection

THP-1 cells were transfected with an individual APP siRNA duplex (GCCTAAAGCTGATAAGAAG) (Dharmacon, Lafayette, CO) (2 × 10^6 ^cells: 2 μg siRNA) using the appropriate Nucleofector program as described by the manufacturer (Amaxa Inc., Gaithersburg, MD).

## Results

**Figure 1:**
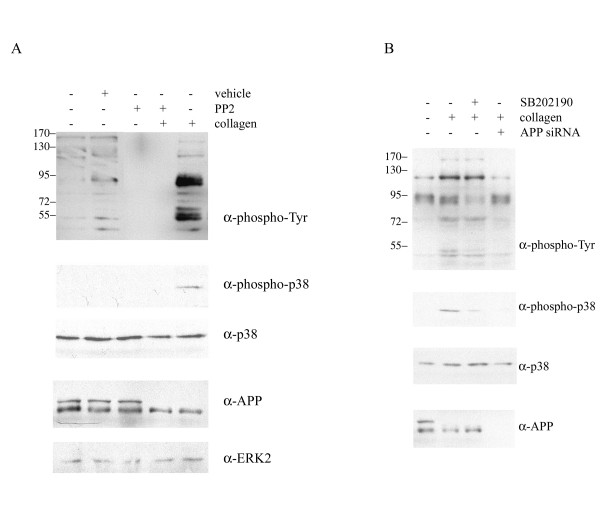
**Adhesion of monocytes to collagen stimulated a tyrosine kinase-dependent signaling response and a tyrosine kinase-independent decrease in full-length APP protein levels**. A) THP-1 cells were either untreated or treated for 30 minutes with PP2 (5 μM) or DMSO vehicle. Cells were plated on tissue culture plastic alone or on type I collagen. B) THP-1 cells were either not transfected or transfected with individual APP siRNA duplexes. At 24 hours post-transfection, non-transfected cells were unstimulated (control) or stimulated by adhesion to collagen, and transfected cells were stimulated by adhesion to collagen. Cells were plated on tissue culture plastic alone or on type I collagen. Cells were lysed at 30 minutes in RIPA buffer, separated by 7% or 10% SDS-PAGE and Western blotted using anti-phosphotyrosine antibody, 4G10, anti-phospho-p38 antibody, anti-p38 antibody (loading control), anti-APP antibody, and anti-ERK2 antibody (loading control). Antibody binding was visualized by chemiluminescence.

In agreement with our previous studies [7], adhesion of THP-1 monocytes to type I collagen stimulated an APP-associated tyrosine kinase-dependent increase in protein phosphotyrosine levels as well as increased levels of the active, phosphorylated form of the p38 mitogen-activated protein kinase (MAPK) (Fig. 1). Our prior work demonstrated the specificity of this response for p38 MAP kinases compared to ERK or JNK enzymes as well as 30 minutes for the optimal stimulation time to observe these changes [7]. Adhesion to collagen also stimulated a decrease in full-length glycosylated APP protein levels suggesting that activation of a collagen receptor on the monocytes stimulated cellular changes that result in either altered secretase activity, increased APP turnover, or impaired transport (Fig. 1). As expected, APP expression was necessary for the stimulated increase in protein phosphotyrosine and phosphorylated p38 levels since siRNA knockdown of APP protein levels was sufficient to attenuate the adhesion-stimulated increase in both (Fig. 1B). Since tyrosine kinase activation is necessary for downstream activation of the p38 MAPK pathway as well as subsequent increases in proinflammatory protein levels [7], it was next determined whether the adhesion-dependent changes in APP processing were also dependent on this signaling. To address this question, the Src kinase family-specific inhibitor, PP2, and the p38-specific inhibitor, SB202190, were used to pretreat THP-1 cells prior to stimulation with collagen. Drug pretreatment, while effective at attenuating the increase in protein phosphotyrosine levels and active, phosphorylated p38 levels, was not sufficient to alter the adhesion-stimulated decrease in full-length APP protein levels suggesting that this change was independent of tyrosine kinase and p38 MAPK activity (Fig. 1).

**Figure 2:**
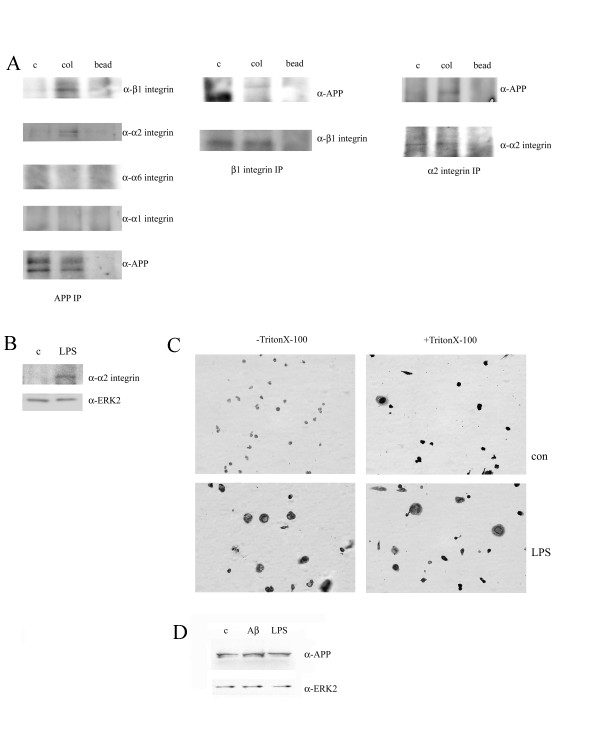
**Ligation of α2β1 integrin with type I collagen stimulated co-localization with APP**. A) THP-1 cells were pre-treated with LPS (25 ng/mL) for 24 hours prior to adhesion to collagen. Cells were plated on tissue culture plastic alone or on type I collagen. Cells were lysed at 30 minutes in 1% Triton X-100 buffer, and either α2 integrin, β1 integrin or APP was immunoprecipitated. Immunoprecipitates were resolved by 7% SDS-PAGE and Western blotted with anti-APP antibody, anti-α1 integrin antibody, anti-α6 integrin antibody, anti-α2 integrin antibody or anti-β1 integrin antibody. B) THP-1 cells were either untreated (c) or treated with LPS (25 ng/mL) for 24 hours. Cells were lysed in RIPA buffer separated by 7% SDS-PAGE and Western blotted using anti-α2 integrin antibody and anti-ERK2 antibody (loading control). C) THP-1 cells were untreated (con) or treated for 24 hours with LPS (1 μg/ml). Cells were plated on coverslips and fixed with 4% paraformaldehyde. Cells were immunostained with and without 1% triton permeabilization with anti-APP antibody. D) THP-1 cells were untreated (c) or treated for 24 hours with LPS (1 μg/ml) or 10 μM Aβ. Cells were lysed in RIPA buffer separated by 7% SDS-PAGE and Western blotted using anti-APP antibody and anti-ERK2 antibody (loading control). Antibody binding was visualized by chemiluminescence.

Previously, we demonstrated that collagen adhesion stimulated formation of a multi-receptor complex including APP and β1 integrin receptors in monocytes and this recruitment of APP to β1 integrins was specific to collagen compared to laminin, fibronectin, and poly-L-lysine [7]. In order to better define this collagen-dependent, β1-APP response and determine which α subunit was involved, immunoprecipitation pull-down assays were performed with and without collagen stimulation to identify specific α receptor recruitment. To increase the probability of detecting APP-integrin receptor association, monocytes were pre-stimulated overnight with LPS which has been demonstrated to upregulate cell surface expression of APP [35,36]. As expected, LPS pretreatment increased cell surface APP levels in the THP-1 monocytes while total protein levels remained the same (Fig. 2C). Additionally, α2 integrin protein levels also increased upon LPS-stimulated activation of these cells (Fig. 2B). Immunoprecipitation with anti-APP antibodies verified that α2 integrin was recruited to a complex with APP and the β1 subunit in contrast to α6 or α1 integrin (Fig. 2A) confirming that a specific APP-β1 integrin receptor complex was formed upon type I collagen interaction. Converse co-immunoprecipitatons using α2 or β1 antibodies confirmed that collagen adhesion stimulated formation of a multi-receptor complex with APP and α2β1 integrins (Fig. 2A).

**Figure 3:**
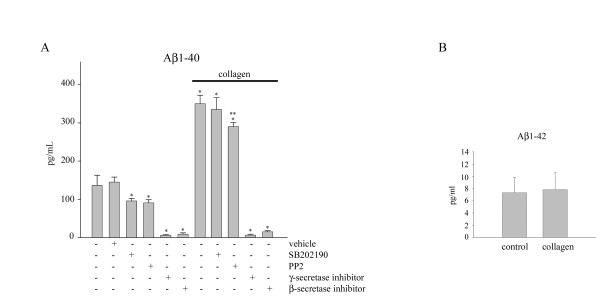
**Collagen stimulates a selective increase in Aβ1-40 that is dependent on Src tyrosine kinase activity**. THP-1 cells were either untreated or treated for 30 min with SB202190 (100 nM), PP2 (5 μM), γ-secretase inhibitor (10 μM), β-secretase inhibitor (5 μM) or DMSO vehicle. Cells were plated on tissue culture plastic or collagen for 48 hours. Media was then collected and analyzed by human Aβ1-40 and Aβ1-42 ELISA. Data were analyzed by unpaired ANOVA with Tukey's post-test comparison and are expressed as mean +/- SD (* = p < 0.001 from control, ** = p < 0.001 from collagen). SB202190 and PP2 treated collagen conditions were normalized to percent decrease due to drug only. Values are representative of three independent experiments.

Monocytes are capable of secreting numerous proinflammatory molecules upon activation, including Aβ [29,37]. Considering these data we hypothesized that adhesion of monocytes to type I collagen would stimulate the release of Aβ. To test this hypothesis, levels of secreted Aβ peptides in the media following monocytic adhesion to collagen were quantitated by ELISA. Monocytes were allowed to adhere to collagen for 48 hours to ensure adequate time to achieve quantifiable levels of Aβ. Adhesion of monocytes to type I collagen stimulated increased secretion of Aβ1-40 and not Aβ1-42 (Fig. 3). To determine if the increased Aβ1-40 release was dependent upon propagation of the tyrosine kinase-dependent signaling or p38 MAPK activity, we again used the tyrosine kinase inhibitor, PP2, or the p38 MAPK inhibitor, SB202190, to inhibit kinase activity in the monocytes prior to and during adhesion. Either inhibitor attenuated basal Aβ1-40 secretion (Fig. 3A). However, when basal inhibition of secretion was used to normalize against the drug effects during collagen stimulated secretion, only pretreatment with PP2 and not SB202190 significantly decreased the release of Aβ1-40 (Fig. 3A). This suggested that a divergence in tyrosine kinase and p38 activation had occurred allowing each to propagate unique phenotypic alteration. However, the dependence of Aβ1-40 secretion on Src family kinase activity is in apparent contradiction with the earlier data demonstrating that tyrosine kinase inhibition was not sufficient to attenuate the collagen-stimulated decrease in full-length APP. As added positive controls for the experiment, cells were treated with either a β secretase or γ secretase inhibitor to verify that direct inhibition of these secretases did indeed attenuate the release of Aβ1-40 (Fig. 3A).

**Figure 4:**
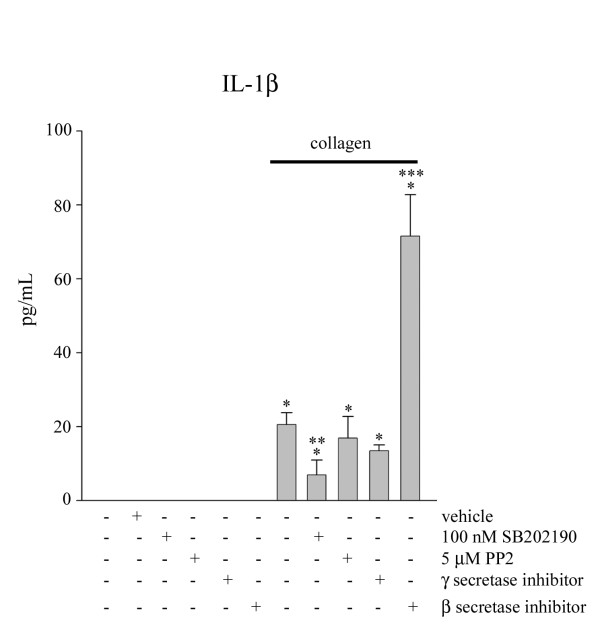
**Adhesion of monocytes to collagen stimulates a p38-dependent increase in IL-1β production**. THP-1 cells were either untreated or treated for 30 min with SB202190 (100 nM), PP2 (5 μM), γ-secretase inhibitor (10 μM), β-secretase inhibitor (5 μM) or DMSO vehicle. Cells were then plated on tissue culture plastic or collagen for 24 hours. Media were collected and analyzed by human interleukin-1β ELISA. Data were analyzed by unpaired ANOVA with Tukey's post-test comparison and are expressed as mean +/- SD (* = p < 0.001 from control, ** = p < 0.01 from collagen, *** = p < 0.001 from collagen). Values are representative of three independent experiments.

In order to further define the APP-α2β1 integrin stimulated phenotype we continued examining adhesion-dependent changes. Specific cytokine secretion, IL-1β, was quantified to determine whether cytokine release was also coupled to the the signaling response. Using commercial ELISA reagents, IL-1β secreted into the media was quantified following a 24 hour stimulation of THP-1 cells with type I collagen in the absence or presence of secretase inhibitors or p38 MAP kinase or Src family kinase inhibitors. In agreement with previous data, there was a significant increase in release of IL-1β from the monocytes following stimulation with type I collagen (Fig. 4). To determine whether release of this proinflammatory molecule was dependent on tyrosine kinase or p38 MAP kinase activity, we used the kinase inhibitors, PP2 and SB202190, respectively, to pretreat the monocytes before and during stimulation with type I collagen. While Aβ1-40 release appeared to be downstream of Src family tyrosine kinase activity (Fig. 3), IL-1β secretion was dependent upon an alternate pathway as it was not affected by tyrosine kinase inhibition but rather on activity of p38 kinase (Fig. 4). Curiously, β secretase inhibition stimulated a robust, significant increase in secreted IL-1β levels only in collagen stimulated cultures and not control cells (Fig. 4).

**Figure 5:**
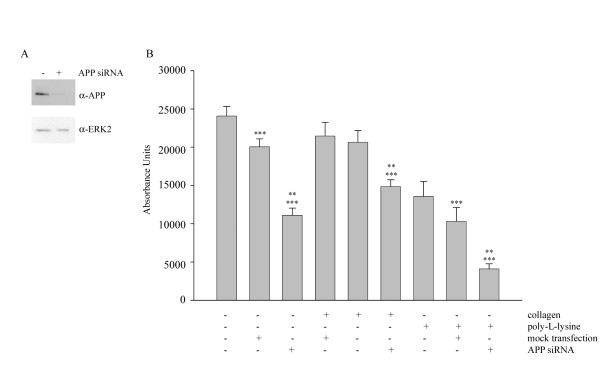
**Adhesion of monocytes to collagen is dependent on expression of APP**. THP-1 cells were either not transfected or transfected with an individual APP siRNA duplex. A) At 24 hours post-transfection, cells were lysed with RIPA buffer, separated by 7% SDS-PAGE and Western blotted with anti-APP antibody and anti-ERK2 antibody (loading control). B) At 24 hours post-transfection, transfected cells and non-transfected cells were loaded with 5 μg/mL calcein-AM and plated on tissue culture plastic alone, poly-L-lysine, or type I collagen for 30 minutes. Fluorescence from total cell number was read prior to washing the wells. Wells were then washed and fluorescence of adhered cells was determined. Data were analyzed by unpaired ANOVA with Tukey's post-test comparison and are expressed as mean +/- SD (*** = p < 0.001 from control, ** = p < 0.001 from mock transfected cells). Values are representative of three independent experiments.

Based upon the observation that APP, and possibly its proteolytic fragments, is recruited to a signaling complex with α2β1 integrin upon cellular interaction with type I collagen and necessary for the complex signaling response and secretory phenotype described above, it was reasonable to assume that APP was necessary for actual adhesion to collagen. As performed previously [7], human APP siRNA transfection was used to decrease APP expression in THP-1 cells (Fig 5A) for comparison to mock transfected cells regarding their ability to bind to type I collagen. Cells that had been transfected to knock-down APP expression versus those which were mock transfected demonstrated significantly reduced adhesion to type I collagen as well as to poly-L-lysine and tissue culture plastic alone (Fig. 5B). This data further verified the role of APP as an adhesion molecule in these cells.

## Discussion

Our data demonstrate that binding of THP-1 monocytes to type I collagen stimulates a tyrosine kinase-dependent proinflammatory signaling cascade that includes activation of p38 MAPK and subsequent increased IL-1β protein levels. In addition, monocyte adhesion to type I collagen stimulates a decrease in full-length glycosylated APP protein levels and a downstream increase in Aβ1-40 release. Furthermore, we have strengthened the existing data that suggest APP itself has a role in adhesion as an APP siRNA-induced decrease in APP protein levels resulted in a significant decrease in adhesion of monocytes to type I collagen.

We previously demonstrated that β1 integrin ligation on monocytes stimulates increased tyrosine kinase activity, recruitment of tyrosine kinases Lyn and Syk to APP, and ultimately a reactive phenotype [7]. β1 integrins form heterodimeric receptors with different α subunits and the specific combination of αβ subunits dictates both binding specificity and overall function of the receptor [38]. Here we identify that it is the α2 subunit that is recruited to the multi-receptor signaling complex during the APP-β1 integrin associated signaling response in monocytes [7]. Therefore, the α2β1 integrin receptor, otherwise known as VLA-2 or CD49b, forms a complex with APP to mediate the proinflammatory activation of THP-1 monocytes upon adhesion to an endogenous ligand, type I collagen. Importantly, both APP and α2 integrin have collagen binding domains in their extracellular regions supporting our observations.

The α2β1 integrin receptor has been identified as a dual collagen/laminin receptor in several cell types [39]. It is a major collagen receptor expressed in keratinocytes, endothelial cells, fibroblasts as well as immune cells. More interestingly, APP and its proteolytic products, have also been suggested to have a role in keratinocyte [40,41], endothelial cell [42,43], fibroblast [44,45] and immune cell [7,36,46,47] function. This correlation supports our observation of a coupled APP-integrin associated function in monocytes but also suggests that this biology will be relevant to a plethora of cell types. It is known that platelets express the necessary secretases to generate Aβ peptides and are suggested to be the major APP expressing cells in the blood [28,48,49]. Moreover, when they are stimulated, including by physiologic agonists such as thrombin and collagen, they are capable of secreting increased levels of various Aβ peptides [50,51]. In turn, platelets can also be stimulated by Aβ1-40 to increase their aggregation [52]. Therefore, platelets have been identified as an important peripheral source of Aβ. Although our study has focused on monocyte APP-α2β1 integrin dependent events, it is possible that this biology will be relevant to platelets as well since this receptor has a prominent role in platelet aggregation.

Integrin-dependent monocyte activation, in particular, involves a complex activation of tyrosine kinases [18]and monocyte adhesion to this extracellular matrix protein stimulates increased cytokine and protease secretion [53-55]. Moreover, it has already been confirmed that α2β1 integrin can operate with other cell surface receptors to mediate its range of effects on phenotype [56,57]. Similarly, our data demonstrated a dependency of α2β1 on APP expression for stimulating type I collagen-dependent increases in tyrosine kinase-dependent signaling and cellular adhesion. Therefore, monocytic functions typically attributed to α2β1 integrin may be altered in animals with decreased or mutant APP expression. For example, studies using α2 knockout animals, have demonstrated an impaired innate immune response[58]. It is intriguing to speculate that APP is also involved in this biology and APP null animals will share a similar dysfunction of innate immune cells.

Besides the APP-α2β1-mediated adhesion of monocytes to type I collagen, we also observed a subsequent, specific processing of APP. Specifically, secreted Aβ1-42 levels remain unchanged after adhesion-mediated activation while secreted Aβ1-40 levels increased. Interestingly, Src family kinase activity regulated the collagen-stimulated increase in secreted levels of Aβ1-40 in spite of the fact that it had no ability to attenuate the decrease in full length glycosylated protein in this stimulation. This implies that tyrosine kinase activity regulates some point in the transport, turnover, secretion or release of Aβ1-40 rather than APP proteolysis. Not surprisingly, Src family kinases have a diverse role in regulating both uptake and release of membrane associated proteins. For example, cell culture observations have demonstrated a role for Src or related family members in positively regulating macropinocytosis [59], negatively regulating kiss and run type exocytosis [60], and positively regulating calveolae release from the plasma membrane through phosphorylation of calveolin-1 [61]. Although the focus of this work has been on Aβ peptide secretion as an endpoint of tyrosine kinase mediated regulation, it is certainly of interest to the laboratory to further define what step kinase activity regulates whether it is transport, proteolysis, or turnover, in future studies.

On the other hand, p38 MAP kinase but not Src family inhibition attenuated an increase in secreted levels of collagen-stimulated release of IL-1β. This data supports the idea that a divergence in the signaling response occurs downstream of the membrane proximal tyrosine kinase activity allowing for discreet regulation of transcriptional and secretory events. Conversely, β secretase but not γ secretase inhibition significantly increased release of IL-1β during collagen stimulation. One interpretation of this result is that Aβ1-40 or some other proteolytic fragment of APP normally acts in an autocrine fashion during collagen stimulation to downregulate the magnitude of proinflammatory activation. However, since tyrosine kinase inhibition had no effect on decreasing IL-1β secretion, yet was able to decrease Aβ1-40 release, it is reasonable to assume that a proteolytic fragment generated after β secretase activity yet before γ secretase activity is involved in downregulating cytokine release. This is in agreement with prior literature demonstrating that exosome secretion of C-terminal fragments of APP can occur *in vitro *suggesting that they may have functions unique from Aβ or APP [62,63]. Indeed, several studies both *in vitro *and *in vivo *have demonstrated that elevations in specific C-terminal fragments, rather than Aβ peptides, mediate cellular death and behavioral dysfunction [64-66]. Tyrosine kinase associated changes have also been linked to unique APP C-terminal fragments, as well. For example, the C99 but not the C89 fragment of APP, upon tyrosine phosphorylation, associates with the adaptor protein, ShcA, to likely propagate a unique signaling response [67].

Although we have not yet determined the consequences of increased monocytic Aβ1-40 secretion following adhesion-stimulated release, there are several possibilities. The secreted peptide could act upon platelets to increase their aggregation [52], stimulate death of endothelium [68] or increase endothelial proinflammatory protein expression to facilitate monocyte transmigration [22,69]. The secreted peptide could also act in an autocrine fashion to stimulate monocytic phenotype changes favoring increased recruitment to the endothelium and subsequent adhesion [70]. This may have some significance with respect to AD pathology as the peptide is able to cross into the brain via interaction with the receptor for advanced glycation end products (RAGE) [71,72], but also may be important in regards to the vascular deposition of Aβ that is also evident in AD [73].

During periods of acute or prolonged peripheral inflammation when there is increased transendothelial migration of monocytes and increased monocytic interaction with extracellular matrix components, this adhesion may specifically perpetuate the inflammatory state by stimulating APP-dependent proinflammatory signaling in the monocytes and secretion of proinflammatory molecules such as IL-1β and Aβ1-40. Indeed, increased monocytic cell surface APP is associated with HIV-associated cognitive impairment [36]) and Alzheimer's disease [35] and cytokine and endotoxin stimulation [35]. Prior studies have reported that monocyte to macrophage differentiation correlates with increased expression of APP [74] consistent with our observations that adhesion dependent activation of monocytes involves APP-integrin dependent signaling. Indeed, others have proposed a similar idea of an APP-related role in phagocyte activation by demonstrating increased expression of gamma secretase components, presenilin and nicastrin, in microglia during the gliotic response after brain injury [75].

## Conclusions

These data suggest that APP has a particular role in regulating monocyte/macrophage immune cell phenotype in response to specific integrin-mediated stimulation. In addition, these findings support the hypothesis that monocyte/macrophage APP may contribute to adhesion-dependent inflammatory changes in both the brain and periphery in varying conditions including Alzheimer's disease, cardiovascular/cerebrovascular disease, and vascular amyloidosis

## Competing interests

The authors declare that they have no competing interests.

## Authors' contributions

CS performed the majority of experiments and data analysis, and wrote the initial version of the manuscript. CC was involved in conceiving the study and coordinating the experiments. He was responsible for editing and revising the manuscript for the final version. Both authors have read and approve the final version of the manuscript.
